# Optimized deep learning model for diagnosing tonsil and adenoid hypertrophy through X-rays

**DOI:** 10.3389/fonc.2025.1508525

**Published:** 2025-03-11

**Authors:** Zhiqing Wu, Ran Zhuo, Yali Yang, Xiaobo Liu, Bin Wu, Jian Wang

**Affiliations:** ^1^ Department of Pediatric Surgery, Children's Hospital of Soochow University, Suzhou, Jiangsu, China; ^2^ Intensive Care Unit, Children's Hospital of Soochow University, Suzhou, Jiangsu, China

**Keywords:** tonsillar, adenoid, artificial intelligence in medicine, ResNet18, YOLOv8, diagnostic imaging

## Abstract

**Objective:**

To explore the application of a deep learning model based on lateral nasopharyngeal X-rays in diagnosing tonsillar and adenoid hypertrophy.

**Methods:**

A retrospective study was conducted using DICOM images of lateral nasopharyngeal X-rays from pediatric outpatients aged 2-12 at our hospital from July 2014 to July 2024. The study included patients exhibiting varying degrees of respiratory obstruction symptoms (disease group). Initially, 1006 images were collected, but after excluding low-quality images and standardizing the imaging phase, 819 images remained. These images were divided into training and validation sets in an 8:2 ratio. The independent test set is consisted of 484 images. We delineated the target areas for tonsils and adenoids and used a YOLOv8n-based model for object detection and use various convolutional neural network models to classify the cropped images, assessing the severity of tonsillar and adenoid hypertrophy. We compared the performance of these models on the training and validation sets using metrics such as ROC-AUC, accuracy, precision, recall, and F1 score.

**Results:**

The combined model, incorporating YOLOv8 for object detection and secondary classification, demonstrated excellent performance in diagnosing tonsillar and adenoid hypertrophy, significantly improving diagnostic accuracy and consistency. The ResNet18 model, due to its lightweight nature and minimal computational resource requirements, performed exceptionally well in the YOLOv8-ResNet fusion model for detecting and classifying tonsils and adenoids, making it our preferred model.

**Conclusion:**

The deep learning model combining YOLOv8n and ResNet18 based on lateral nasopharyngeal X-rays demonstrates significant advantages in diagnosing pediatric tonsillar and adenoid hypertrophy.

## Introduction

Tonsillar and adenoid hypertrophy are common upper respiratory tract diseases in children, significantly affecting their health and quality of life. The adenoids and tonsils are important lymphoid tissues in the pharynx, responsible for filtering pathogens entering the respiratory tract. However, when these tissues become excessively hypertrophic, they can cause symptoms such as mouth breathing, nasal obstruction, difficulty breathing, snoring, and sleep apnea. If left untreated, this can lead to facial deformities, growth retardation, cognitive impairment, increased cardiovascular risk (1), and behavioral problems (2). Adenoid and tonsillar hypertrophy are independently associated with the risk of pediatric obstructive sleep apnea syndrome (OSAS), with the prevalence of OSAS increasing with the size of the adenoids and tonsils (3). Adenotonsillectomy and medication are common treatments for children with adenoid and tonsillar hypertrophy who suffer from OSAS.

**Figure 1:**
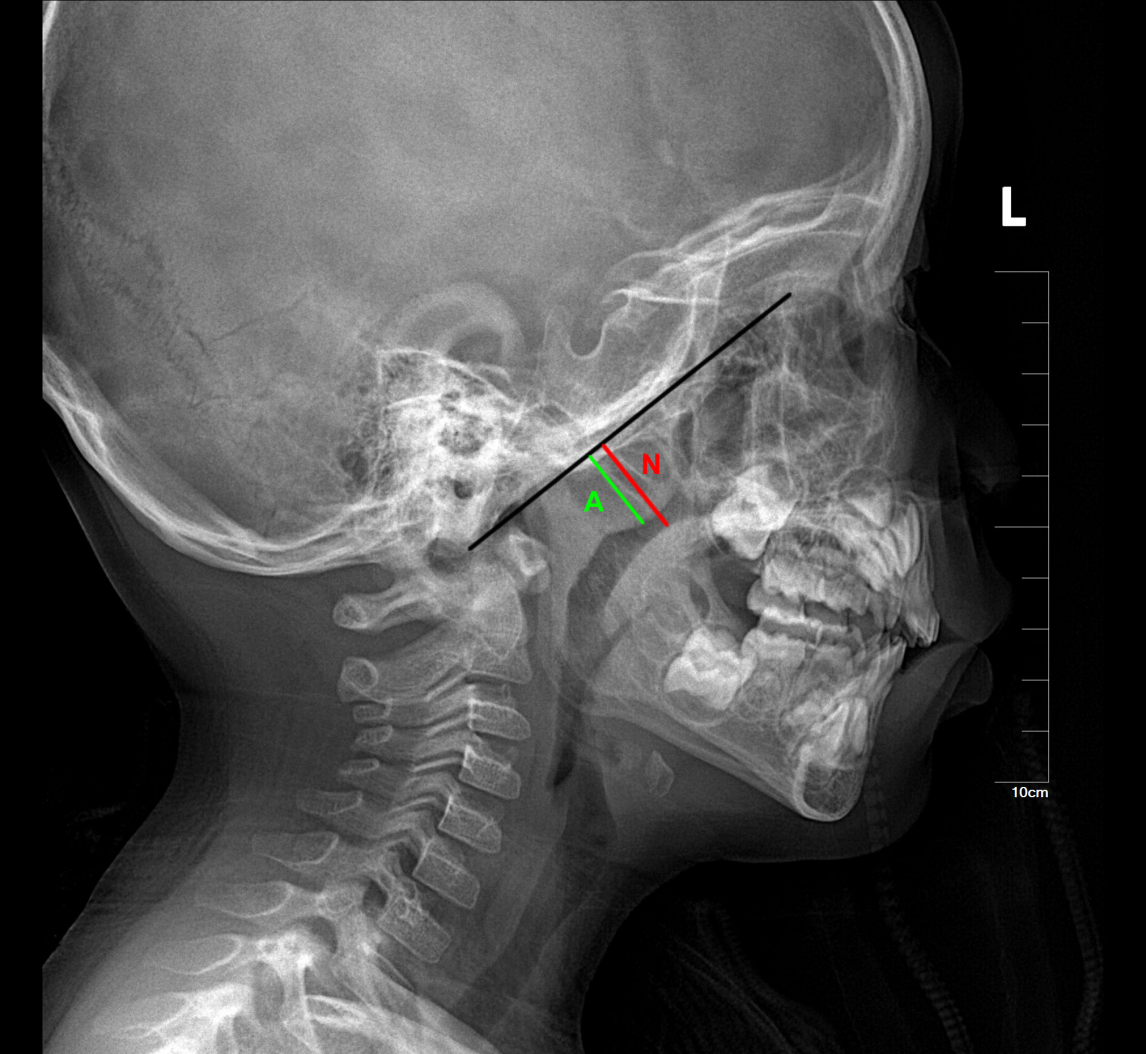
The Fujioka method for measuring adenoid size on lateral radiographs by calculating the adenoid/nasopharyngeal (A/N) ratio. The black line marks the lateral contour of the occipital slope of the skull on the lateral radiograph. The green line (A) represents the vertical distance from the most prominent point of the adenoid to the lateral contour of the occipital slope of the skull. The red line (N) represents the width of the nasopharyngeal cavity at the level of the most prominent point of the adenoid.

Lateral cephalometric radiographs, a standard orthodontic method for evaluating craniofacial morphology, provide orthodontists with readily available references for assessing airway obstruction and hypertrophic adenoids and tonsils (4). Numerous studies have reported a reasonable correlation between cephalometric measurements of adenoids and their size (5). Lateral cephalometric radiographs are accurate in diagnosing adenoid hypertrophy (6), but there is a lack of sufficient guidelines for diagnosing tonsillar hypertrophy using cephalometric measurements (7). Currently, the Fujioka method (8) is used to measure adenoid size on lateral radiographs by calculating the adenoid/nasopharyngeal (A/N) ratio (**Figure 1**). This ratio is derived by dividing the adenoid measurement (A) by the distance from the posterior edge of the hard palate to the anterior inferior edge of the spheno-occipital junction (N). An A/N ratio greater than 0.67 is considered indicative of adenoid hypertrophy.

The standard grading system for diagnosing tonsillar size and hypertrophy is based on clinical oropharyngeal examination (9), grading tonsils by the percentage they occupy in the oropharyngeal airway’s diameter (10). However, this widely used method is imperfect as it cannot show anterior-posterior obstruction of the oropharynx (11–13). Some literature suggests using the T/O ratio to evaluate tonsillar hypertrophy (7), but these previous evaluation metrics have certain limitations (14). They are also relatively cumbersome to operate and require a high level of diagnostic and assessment proficiency from different physicians.

In recent years, deep learning has made significant progress in medical image analysis and diagnosis (15), with broad applications in image segmentation for trauma fractures (16) and lung nodule diagnosis (17). This paper proposes a deep learning method combining YOLO (You Only Look Once) object detection and image classification for diagnosing tonsillar and adenoid hypertrophy in lateral nasopharyngeal X-rays. Tonsillar and adenoid hypertrophy are common pediatric health issues that can lead to airway obstruction, sleep apnea syndrome, and recurrent infections, among other serious health problems. Traditional diagnostic methods rely on the clinical experience of physicians, which can be subjective and have limitations in diagnostic accuracy. In contrast, deep learning models can reduce diagnostic bias (18). Therefore, developing an automated computer-aided diagnostic method is of paramount importance. Currently, there is no research that utilizes a combination of YOLO for object detection and CNNs for secondary classification to diagnose tonsillar and adenoid hypertrophy on the same lateral nasopharyngeal X-ray images.

## Materials and methods

### Materials preparation

**Figure 2:**
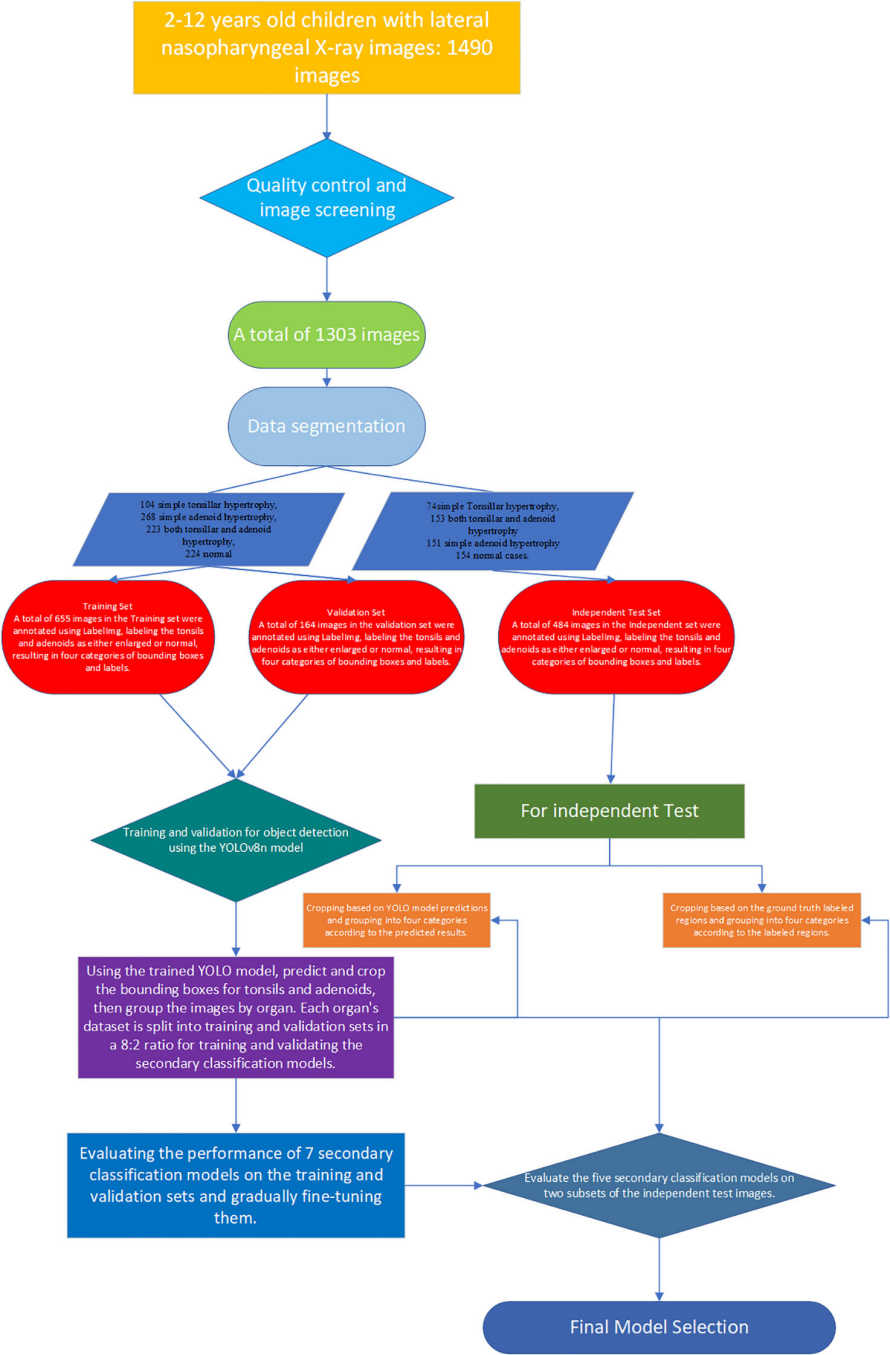
Flowchart of data collection and construction of a deep learning integrated model.

Tonsils and adenoids reach their maximum size at around ages 7-9 and 12-13, respectively (19). In this study (**Figure 2**), we included children aged 2-13, who are the most common candidates for adenoidectomy and tonsillectomy. We collected lateral nasopharyngeal X-ray DICOM images of pediatric outpatients from our hospital from July 2014 to July 2024. These images were used as training, validation, and independent test sets. Initially, there were 1006 images in the training and validation sets, which were reduced to 819 images after excluding low-quality images and standardizing the imaging phase. The validation set included 164 images, evenly distributed among 104 cases of simple tonsillar hypertrophy, 268 cases of simple adenoid hypertrophy, 223 cases of both tonsillar and adenoid hypertrophy, and 224 normal cases. An additional independent test set was collected, consisting of 484 images, including 74 cases of simple tonsillar hypertrophy, 153 cases of both tonsillar and adenoid hypertrophy, 151 cases of simple adenoid hypertrophy, and 154 normal cases. The inclusion criteria for the collected images were: (1) adequate radiographic image quality, (2) children in the disease group with varying degrees of respiratory obstruction symptoms, and (3) children in the normal group without any clinical symptoms. Exclusion criteria included: (1) poor image quality or low contrast blurry images, (2) abnormal postures of the children during imaging (e.g., excessive neck extension or flexion), and (3) images taken in the inverse phase. The images were initially diagnosed by radiologists with 3 years of experience, and the final diagnoses were confirmed by two attending radiologists with 5-8 years of experience. Any discrepancies in diagnoses were resolved through discussion.

This study obtained parental consent and was approved by the Institutional Ethics Committee of our hospital.

### Research methods

**Figure 3:**
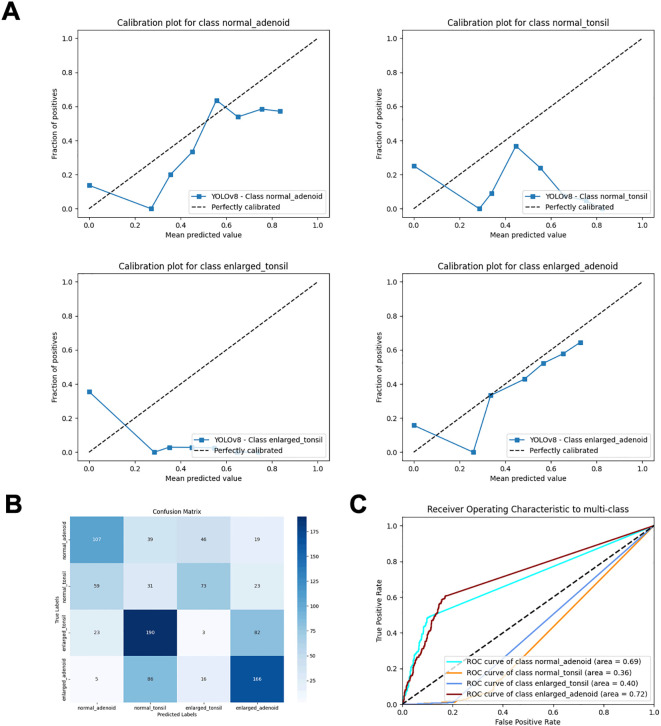
Performance metrics of direct object detection and inference classification using the YOLOv8 model. **(A)** Calibration plots for each class. **(B)** Confusion matrix. **(C)** ROC curves.

YOLOv8 (20) (You Only Look Once) models use Darknet-53 (21) as their backbone, which includes residual and convolutional blocks. This model transforms object detection into a regression problem by directly predicting the class and location of the object. Previous studies have utilized this model for facial landmark detection (22), and Wenting Xie et al. applied an improved YOLOv8 model for ovarian cancer diagnosis (23). While YOLO models perform well in object detection and localization, they may not be as effective in distinguishing different states of the same organ (**Figure 3**), particularly in medical imaging. CNN models, composed of multiple stacked convolutional layers, are advanced deep learning technologies. They typically consist of an input layer (image input), a convolutional layer (which convolves the input image with filters to generate feature maps), a Rectified Linear Unit (ReLU) activation layer (which activates neurons above a threshold), a pooling layer (which reduces image size while retaining high-level features), and a fully connected (FC) layer (which produces the final results). These models can automatically extract features from image data and classify the images, and have been applied to various radiology tasks, achieving high performance in image-based disease classification. We found that combining CNN models with YOLO models can enhance the classification performance, especially in distinguishing subtle features. This approach integrates feature extraction and classification within the same network, providing a streamlined and efficient process. However, CNNs can struggle with imbalanced datasets, which often requires various techniques to balance the data. Data augmentation is commonly used in the medical field to increase the size of datasets. This method generates additional labeled images without altering the semantics of the images, thereby mitigating dataset imbalance. In this study, we used several data augmentation methods, such as gamma transformation and horizontal flipping, which do not affect the vertical orientation of the nasopharyngeal X-rays. Although CNNs require large labeled medical datasets for training, which can be challenging to create due to time and labor costs, recent research suggests that transfer learning can be a solution for small dataset issues.

In transfer learning (TL), convolutional neural networks (CNNs) are first trained to learn features from a broad domain, such as ImageNet. The trained features and network parameters are then transferred to a more specific domain. In CNN models, low-level features like edges, curves, and corners are learned in the initial layers, while more specific high-level features are learned in the final layers. Among various TL models, we selected seven CNN models, including ResNet18, ResNet34, ResNet50, DenseNet121, EfficientNet-B0, VGG16, and AlexNet, to compare their performance using different metrics. Ultimately, we chose to combine the ResNet18 with the YOLOv8n model. ResNet-18 is a convolutional neural network with 18 layers that addresses the issue of training deep networks by introducing residual blocks. Its architecture includes an initial convolutional layer, four stages of residual blocks (each stage containing two 3x3 convolutional layers), a global average pooling layer, and a fully connected layer. This design allows the network to effectively train deep models while maintaining relatively low computational complexity. A recent study (24) evaluated the performance of several neural networks with a softmax output layer and ReLU activation, confirming the superior performance of softmax with ReLU in classification tasks. Therefore, to obtain probabilistic predictions, we used a fully connected layer as the output layer and modified it for four-class classification. We chose the cross-entropy function as the loss function, which inherently includes the Softmax operation, eliminating the need for an explicit softmax definition. The structure of ResNet18 incorporates a key design feature known as residual blocks. These blocks allow the model to use ‘skip connections’ to pass inputs directly to subsequent layers, bypassing some intermediate layers of neurons. This design helps to mitigate the vanishing and exploding gradient problems that can arise as the network depth increases, which makes it easier for the network to learn deep representations without degradation. Through this structure, ResNet18 can effectively capture both low-level and high-level features in images, leading to excellent performance in image classification tasks. In the process of transfer learning and fine-tuning, we initially froze the first four modules of the model and gradually unfroze all layers during training. During training, we calculated the output and loss through forward propagation and updated the weights via backpropagation. We also dynamically adjusted the learning rate to avoid catastrophic forgetting. Additionally, we modified the output of the fully connected (FC) layer to accommodate a four-class classification task. After testing various optimizers, we selected the “Adam” optimizer for its superior performance among all those studied. Consequently, we applied this optimizer during the model training process. To understand the model’s attention to different regions of the images, we employed a visualization tool called Gradient-weighted Class Activation Mapping (2D-Grad-CAM) (25) to analyze the interpretability of the five fused deep learning models. This tool visually identifies the correspondence between the regions of interest in the pathological area and the model’s prediction attention. In this study, we focused on whether the models not only paid attention to the tonsil and adenoid areas but also evaluated the regions of the airway.

The collected lateral nasopharyngeal X-rays were first converted from DICOM files to JPG images using Python’s PIL package. Then, experienced radiologists annotated the target detection boxes using the LabelImg software, marking the tonsil and adenoid detection regions on each X-ray. The annotations were completed collaboratively by two experts. For images where there was disagreement, the experts engaged in discussions to reach a consensus and ensure the accuracy of the classifications. The adenoid region was defined from the upper edge of the nasopharynx to the palatal plane, from the posterior edge of the hard palate to the posterior pharyngeal wall and the anterior aspect of the C1 vertebra. The tonsil detection region was defined from the palatal uvula level to the posterior aspect of the tongue, from the anterior aspect of the C3 vertebra to the aryepiglottic fold. The detection boxes were rectangular regions determined by vertical and horizontal lines passing through these anatomical landmarks, ensuring that the entire airway cross-sectional content of the tonsil or adenoid was included in the training images and improving the localization accuracy of the YOLO model’s detection boxes.

After defining the detection boxes, we devised a two-stage detection + diagnosis method to diagnose whether children had tonsillar or adenoid hypertrophy on the same lateral nasopharyngeal X-ray. First, we fine-tuned the pre-trained YOLOv8m model on ImageNet to learn the detection box locations, adjusting model parameters and hyperparameters through iterative training. After 135 epochs, we controlled the box loss error range within 0.5%, using IOU-based evaluations to improve detection accuracy. The model’s final weights were saved corresponding to the lowest box loss during the last training iteration. For the training and evaluation of the secondary classification model, we first used the trained YOLO model to predict and crop the original images from the training and validation sets. The highest-confidence detection boxes for the predicted tonsil and adenoid regions were selected as the final detection boxes. These final detection boxes were then used to crop the original images, resulting in predicted cropped images. These cropped images were categorized into four classes: normal tonsils, enlarged tonsils, normal adenoids, and enlarged adenoids. We then re-established the training and validation sets, and the cropped images were fed into the secondary classification models, pretrained on ImageNet, to learn image features for classification. This process generated the optimal model weight files for different base models and organs.

During the evaluation on the independent test set, we repeated the same procedure, using the secondary classification models to predict and obtain the labels for the cropped images. These predicted labels were compared with the true labels for the independent test set. We utilized two methods for correction: first, we evaluated the predicted cropped images and true labels from the independent test set; second, we cropped the original images using the true labels and input these into the secondary classification models for performance evaluation.

The results from these two evaluation methods were used to assess the secondary classification models, determining the optimal models for classifying tonsil and adenoid images. This approach ensured the overall performance of the combined YOLO model and secondary classification model was optimal.

### Statistical analysis

We use the following indicators to evaluate the performance of the model and select the best model: ROC AUC,accuracy, sensitivity, specificity, F1 score, Confusion Matrix Visualization images. This study used the following tools: Python 3.7.16 (https://www.python.org/downloads/release/python-3716/) and PyTorch third-party libraries(Version: 1.13.1)on Windows 11 operating system ([MSC v.1916 64 bit (AMD64)]).

## Results

**Figure 4:**
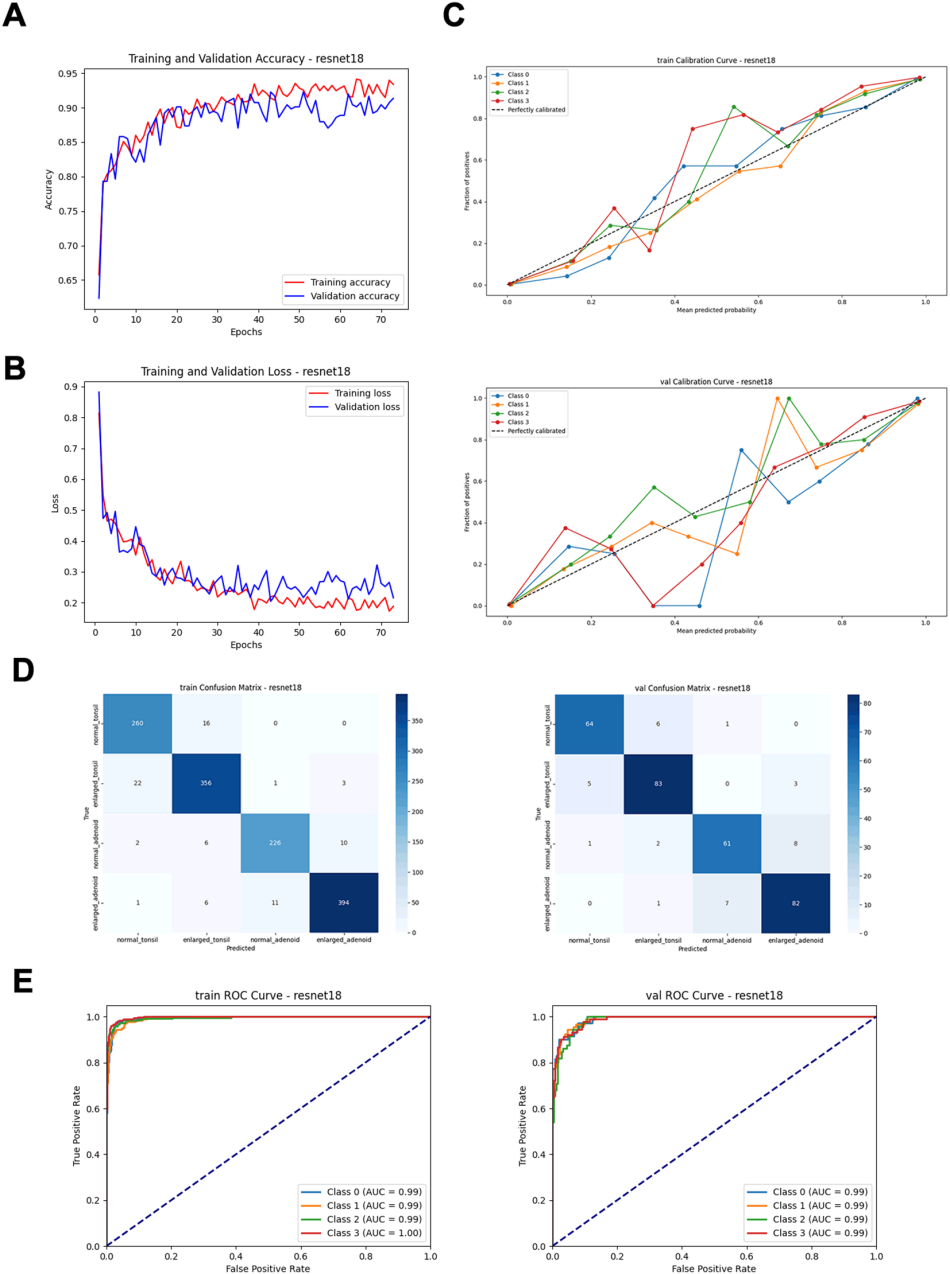
Performance metrics of the ResNet18 model on training and validation sets. **(A)** Training and validation accuracy curves. **(B)** Training and validation loss curves. **(C)** Calibration curves. **(D)** Confusion matrices. **(E)** ROC curves.

**Figure 5:**
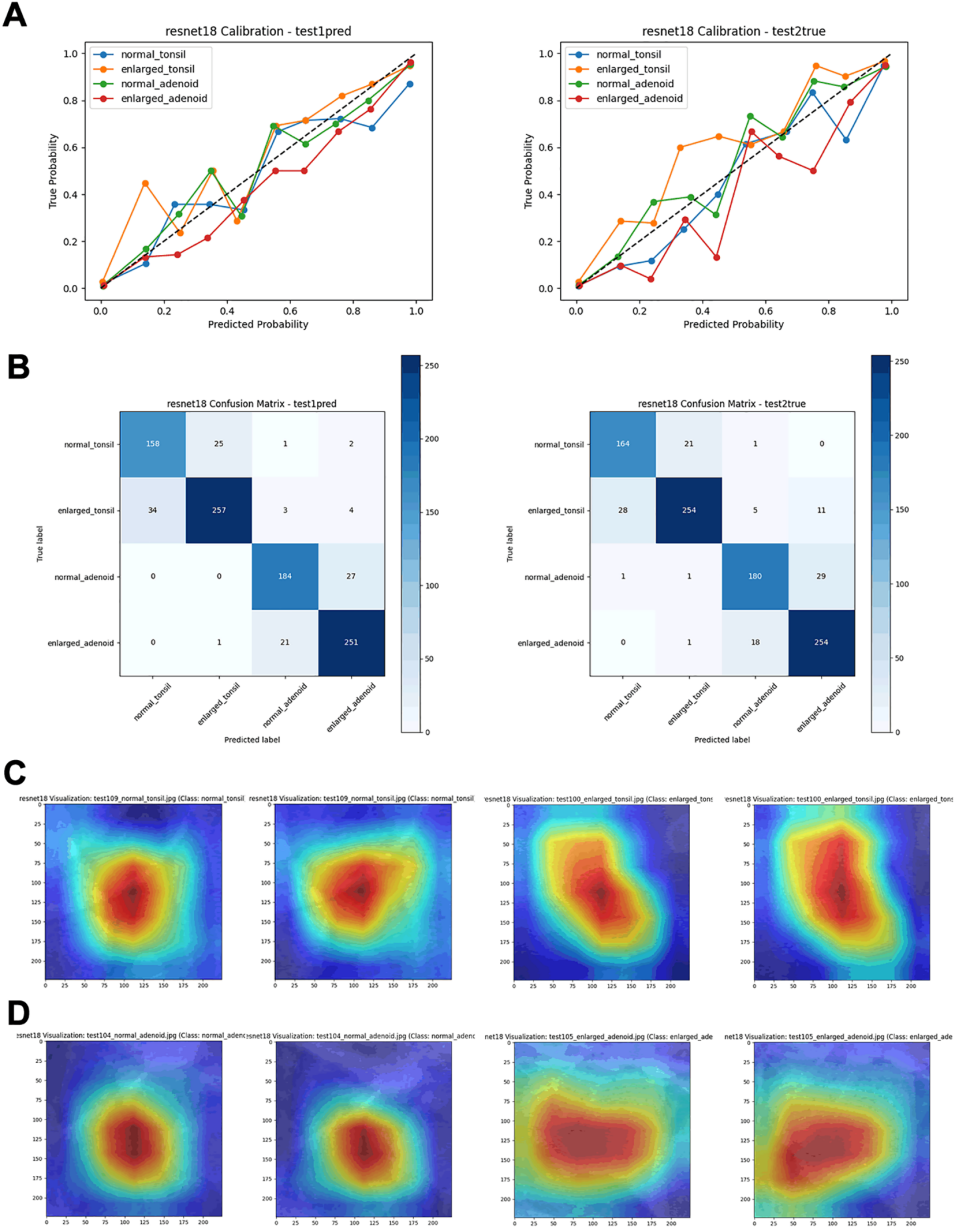
Performance metrics of the ResNet18 model on test sets. **(A)** Calibration curves. **(B)** Confusion matrices. **(C)** Visualization heatmaps of the tonsil region. **(D)** Visualization heatmaps of the adenoid region.

By comparing performance metrics across the training, validation (**Figure 4**, **Table 1**), and independent test sets (**Figure 5**, **Table 2**), along with visualizing confusion matrices and the regions of interest highlighted by the models, we concluded that the fusion model consisting of YOLOv8n as the front-end model for object detection and localization, combined with the fine-tuned ResNet18 as the back-end secondary classification model, demonstrates significant advantages in diagnosing conditions using lateral nasopharyngeal X-ray images.

**Table 1:** Performance metrics of different secondary models on training and validation sets.

Model_Name	Phase	Roc_Auc	Accuracy	Precision	Recall	F1	Logloss
resnet18	train	{0: 0.995, 1: 0.993, 2: 0.994, 3: 0.997}	0.941	0.941	0.941	0.941	0.169
	val	{0: 0.990, 1: 0.990, 2: 0.986, 3: 0.989}	0.895	0.895	0.895	0.895	0.250
resnet34	train	{0: 0.994, 1: 0.994, 2: 0.998, 3: 0.997}	0.936	0.936	0.936	0.936	0.152
	val	{0: 0.993, 1: 0.992, 2: 0.981, 3: 0.991}	0.926	0.927	0.926	0.926	0.239
resnet50	train	{0: 0.996, 1: 0.995, 2: 0.994, 3: 0.996}	0.935	0.935	0.935	0.935	0.157
	val	{0: 0.993, 1: 0.993, 2: 0.982, 3: 0.991}	0.907	0.908	0.907	0.907	0.238
densenet121	train	{0: 0.907, 1: 0.876, 2: 0.885, 3: 0.939}	0.693	0.692	0.693	0.686	0.776
	val	{0: 0.932, 1: 0.897, 2: 0.907, 3: 0.937}	0.698	0.713	0.698	0.691	0.759
efficientnet_b0	train	{0: 0.897, 1: 0.871, 2: 0.845, 3: 0.912}	0.666	0.683	0.666	0.655	0.848
	val	{0: 0.938, 1: 0.879, 2: 0.857, 3: 0.870}	0.630	0.663	0.630	0.601	0.908
vgg16	train	{0: 0.967, 1: 0.964, 2: 0.965, 3: 0.977}	0.824	0.825	0.824	0.824	0.412
	val	{0: 0.969, 1: 0.961, 2: 0.966, 3: 0.970}	0.815	0.823	0.815	0.812	0.452
alexnet	train	{0: 0.968, 1: 0.959, 2: 0.963, 3: 0.974}	0.828	0.829	0.828	0.828	0.422
	val	{0: 0.976, 1: 0.955, 2: 0.969, 3: 0.966}	0.812	0.814	0.812	0.812	0.432

**Table 2:** Performance metrics of different secondary models on test sets.

Model_Name	Phase	Accuracy	Precision	Recall	F1 Score	Log Loss	ROC AUC	Cohen Kappa	Matthews Correlation Coefficient
resnet18	Test1	0.878	0.879	0.878	0.878	0.389	0.975	0.836	0.836
	Test2	0.880	0.882	0.880	0.880	0.345	0.979	0.838	0.839
resnet34	Test1	0.871	0.872	0.871	0.871	0.453	0.972	0.826	0.826
	Test2	0.872	0.875	0.872	0.871	0.368	0.981	0.827	0.829
resnet50	Test1	0.851	0.858	0.851	0.849	0.468	0.974	0.798	0.801
	Test2	0.867	0.875	0.867	0.864	0.421	0.978	0.819	0.823
densenet121	Test1	0.415	0.555	0.415	0.350	1.260	0.814	0.187	0.257
	Test2	0.403	0.548	0.403	0.336	1.295	0.808	0.171	0.238
efficientnet_b0	Test1	0.406	0.457	0.406	0.358	1.219	0.763	0.181	0.216
	Test2	0.440	0.516	0.440	0.395	1.171	0.783	0.227	0.268
vgg16	Test1	0.851	0.853	0.851	0.851	0.397	0.973	0.799	0.800
	Test2	0.857	0.860	0.857	0.856	0.367	0.978	0.807	0.809
alexnet	Test1	0.844	0.848	0.844	0.842	0.429	0.972	0.788	0.791
	Test2	0.845	0.855	0.845	0.844	0.399	0.978	0.790	0.794

In this experiment, we explored the use of YOLOv8n for object detection and localization, combined with various deep learning models like ResNet18 for secondary classification. YOLOv8n accurately identifies the regions of interest, while the subsequent classification is performed by models such as ResNet18. The goal of this approach was to improve classification accuracy for four categories: normal adenoid, normal tonsil, enlarged adenoid, and enlarged tonsil. The results showed that the combination of YOLOv8n and ResNet18 performed exceptionally well across several key metrics. On the independent test set, our combined model achieved an impressive classification accuracy of 97%, with AUC values above 0.95 for each category. These results were further corroborated by the ROC curves, which demonstrated strong discriminatory power between the different classes. While other models like AlexNet, VGG16, DenseNet121, EfficientNet-B0, ResNet34, and ResNet50 also performed well, especially in certain complex classification tasks, they generally fell short of ResNet18’s performance. Despite having ROC AUC values close to or reaching 0.95, the confusion matrices and calibration curves revealed that these models had slightly higher misclassification rates and calibration inaccuracies in some categories. In particular, DenseNet121 and EfficientNet-B0, while stable in classification tasks, showed some limitations in fine-grained classification compared to ResNet18. In summary, through a comprehensive analysis of the classification reports, ROC curves, calibration curves, and confusion matrices across the training, validation, and independent test sets, we found that the combination of YOLOv8n and ResNet18 outperformed the other models in this four-classification task. This model structure not only improved classification accuracy and stability but also demonstrated good generalization capability, making it suitable for practical applications. Therefore, we ultimately chose the YOLOv8n and ResNet18 fusion model as the integrated approach for diagnosing tonsil and adenoid enlargement based on lateral nasopharyngeal radiographs. Currently, the treatment standards for tonsillar and adenoid hypertrophy are based on the presence of clinical symptoms. For patients with clinically significant symptoms or recurrent inflammation due to hypertrophic tonsils or adenoids, tonsillectomy and adenoidectomy are recommended. Our deep learning model can play a role in large-scale screenings and help avoid unnecessary surgical interventions caused by diagnostic variability among individuals.

## Discussion

### Diagnostic approach

Lateral nasopharyngeal X-rays offer convenience, low cost, high repeatability, and ease of operation. Compared to maxillofacial CT scans (14), they have a lower radiation dose, making them less harmful to children. Additionally, they provide better standardization and operability compared to ultrasound examinations, which often depend on the skill and subjective judgment of the ultrasonographer and lack the standardization of nasopharyngeal X-rays (26, 27). Currently, clinical practice often involves diagnosing based on nasopharyngeal X-rays combined with clinical symptoms. We selected children who already exhibited symptoms and underwent X-ray imaging to ensure diagnostic accuracy.

Previous studies on deep learning have primarily focused on the diagnosis of adenoid enlargement, without including tonsillar hypertrophy. In contrast, our study addresses the diagnosis of both tonsillar and adenoid enlargement. Many of the earlier studies on diagnosing adenoid enlargement using X-ray nasopharyngeal lateral films relied on manual annotation (28), which could lead to inconsistent diagnostic quality due to variability in annotation skills, and the process is often time-consuming. Some studies have used CT images for automated adenoid enlargement diagnosis (29); however, CT scans involve higher radiation doses than X-rays, making them less suitable for routine screening. More recent studies have employed region-of-interest (ROI) delineation followed by deep learning models for automated diagnosis (30), but this still requires preprocessing of the X-ray nasopharyngeal lateral films. Other research has focused on detecting anatomical landmarks on nasopharyngeal lateral images to automatically diagnose adenoid enlargement (31–33). Although these methods have demonstrated strong diagnostic performance, they are less intuitive in their presentation and still require preprocessing of the nasopharyngeal lateral films.

### Reducing error transmission impact

In this stepwise model architecture, errors in the target detection phase can propagate to the classification phase. If YOLOv8 fails to accurately locate the target area or mistakenly detects background regions, the subsequent classification model will process incorrect input data, reducing classification accuracy. To mitigate this impact, we corrected the performance evaluation results using real cropped images during the evaluation phase, ensuring accurate prediction and classification by the model.

### Handling complex scenarios

In medical images, the positions and shapes of tonsils and adenoids may vary due to patient differences and imaging angles. YOLOv8, as a powerful object detection tool, can adapt to these variations and accurately locate targets, which is crucial for handling complex scenarios and ensuring precise localization. The subsequent convolutional neural network can then perform fine-grained classification based on these accurate localization results. This ability to handle complex scenarios enhances the robustness and reliability of the entire system in practical applications.

### Limitations of our study

This study has several limitations. It is a single-center study with a relatively small sample size, and the sample type (lateral nasopharyngeal X-rays) limits the amount of information that can be obtained from the images. Additionally, the 8:2 ratio for training and validation sets results in a small number of validation images. Accurate diagnosis and treatment of tonsillar and adenoid hypertrophy require consideration of clinical symptoms, and relying solely on X-rays is inappropriate. Although Resnet18 performed well compared to other models, the accuracy curve showed some fluctuations in the later stages of training. An ideal model would have high and closely aligned accuracy curves, ensuring both fitting capability and generalization ability. This fluctuation might indicate variable performance during each training epoch, especially when using small batch sizes.

In the future, we plan to expand the sample size, closely monitor changes in loss and accuracy curves, and collaborate with multiple centers to collect more lateral nasopharyngeal X-rays. This will increase the number of images in the training and validation sets and add additional test sets to validate the model’s prediction ability on unseen images, aiming to develop a more accurate disease diagnosis model. We will also explore using more advanced front-end models to replace YOLOv8 for target detection, further reducing error transmission. Additionally, we will attempt to use more lightweight models and optimize them for operation on mobile devices, making the model more convenient and effective for use in primary healthcare settings.

## Conclusion

The deep learning model combining YOLOv8n and ResNet18 based on lateral nasopharyngeal X-rays demonstrates significant advantages in diagnosing pediatric tonsillar and adenoid hypertrophy.

## Data Availability

The original contributions presented in the study are included in the article/**Supplementary Material**. Further inquiries can be directed to the corresponding author.
